# Osteopathic Approach for Keloids and Hypertrophic Scars

**DOI:** 10.7759/cureus.44815

**Published:** 2023-09-07

**Authors:** Bruno Bordoni, Allan R Escher, Gregory T Girgenti, Filippo Tobbi, Roberto Bonanzinga

**Affiliations:** 1 Physical Medicine and Rehabilitation, Foundation Don Carlo Gnocchi, Milan, ITA; 2 Anesthesiology/Pain Medicine, H. Lee Moffitt Cancer Center and Research Institute, Tampa, USA; 3 Anesthesiology, H. Lee Moffitt Cancer Center and Research Institute, Tampa, USA; 4 Osteopathy, PGO (Post Graduate Osteopathic) Institute, Lesignano De' bagni, ITA

**Keywords:** fascia, manual therapy, osteopathy, osteopathic manipulation, atrophic scar, striae distensae, hypertrophic scar, keloid

## Abstract

The skin is a complex organ, a system that influences and is influenced by the body system, with different skin layers always mechano-biologically active. In the presence of a lesion that damages the dermis, the skin undergoes sensory, morphological, and functional alterations. The subsequent adaptation is the formation of scar tissue, following distinct and overlapping biological phases. For reasons not yet fully elucidated, some healing processes lead to pathological scars, from which symptoms such as pain, itching, and functional limitations are derived. Currently, there is no gold standard treatment that fully meets the needs of different scars and can eliminate any symptoms that the patient suffers. One such treatment is manual medicine, which involves direct manual approaches to the site of injury. Reviewing the phases that allow the skin to be remodeled following an injury, this article reflects on the usefulness of resorting to these procedures, highlighting erroneous concepts on which the manual approach is based, compared to what the current literature highlights the cicatricial processes. Considering pathological scar adaptations, it would be better to follow a gentle manual approach.

## Introduction and background

The skin that covers the human body is one of the largest and most complex organs, with an area of about 1.8 square meters and an average thickness of 0.3 cm [1]. The skin has a double phylogeny. The epidermis derives from the ectoderm; in animal models, the epidermis appears around the ninth day of gestation through the expression of the growth factor p63, fundamental for the epidermal lineage [2,3]. This most superficial layer is composed of further layers, such as the corneum, granular, stratum spinous, stratum lucidum (found only in the soles of the feet and palms of the hands), and stratum basale or stratum germinativum [1,2].

The integrity of the developed epidermis is constantly managed by proliferative and undifferentiated epidermal stem cells (residing in the basal layer), which are essential for the maintenance of different skin functions [2]. The dermis has a more complex development, as for the craniofacial area and part of the cervical tract it derives from the mesoderm and neural crests (ectoderm), while for the remaining body area, it derives from the somites (mesoderm), around the day 10-15 of gestation (animal model) [4]. The dermis is about 10 times thicker than the epidermis. Dermal stem cells are mainly found in the connective tissue dermal sheath and in the hair follicle dermal papilla [5]. We can distinguish two layers, such as the papillary and the reticular dermis [6].

The hypodermis is the layer below the dermis, the site of adipose tissue [5]. During embryogenesis, the WNT-11 (Wnt Family Member 11) protein plays an important role in the orientation of the dermal lineage [7]. The most representative cells of the epidermis, the keratinocytes, will derive from the basal layer of the epidermis; the latter, through the presence of Toll-like receptors, recognize microbial substances and secrete microbial peptides, such as defensins and cathelicidins [8]. The epidermis is the first line of defense of the immune system. We can find other cells, such as melanocytes (for skin pigmentation), Merkel's cells (mechanoreceptors), and Langerhans' cells (defense against antigens) [8]. In the dermis, we find collagen and fibroblasts, nerve endings, glands, small blood vessels, and follicles [6]. In the papillary dermis, there are Schwann cells or glial cells, which are mechanosensitive and connected to unmyelinated nociceptive nerves; their endings reach the epidermis and can carry nociceptive information [6,9]. The reticular dermis allows the dermal layer to be elastic and withstand mechanical stresses [6].

The integumentary system is the first defensive barrier against external stresses. The skin can repair injured areas, for example, due to trauma or surgery, through complex local and systemic mechanisms. The restoration and filling of the skin layers will not have the original physiological characteristics, in particular, if the lesion reaches the dermis [10,11]. The healing processes can follow aberrant patterns and result in hypertrophic and keloid scars. The detection rate of hypertrophic scars can be up to 72% of all people treated in hospitals (100 million people are treated in hospitals each year) [10]. The rate of keloid formation is highly dependent on skin pigmentation, with a finding of about <0.1% for people with low pigmentation and a maximum of 10% for people with high pigmentation [12]. The article reviews the physiological and pathological healing processes leading to wound resolution or symptomatic scar formation, respectively. Furthermore, the article raises reflections on what a manual approach to scars could be, which is as useful as possible.

## Review

Repair phase: homeostasis

The wound-healing phase involving the dermis and epidermis begins with the homeostasis phase. Blood clots made up of platelets, fibrins, and fibronectins begin to form the first barriers to limit blood loss and fight any infections, with a concomitant contraction of the blood vessels [10,13,14]. The smooth muscle cells of the damaged vessels are stimulated by endothelins (vasoconstrictor substance) to vasoconstriction [10]. In this first phase of homeostasis, the platelets change shape (the cytoskeletal actin is deformed) from round to flattened, to interact better with the integrins of the extracellular matrix [10]. The second phase of the homeostasis process, thanks to platelet aggregation, is characterized by the activation of coagulation processes. The enzyme or factor Xa (arginine-specific serine protease) is activated, which stimulates prothrombin 1 and 2 and meizothrombin; from prothrombin 2, the synthesis of thrombin will be reached, while the other two enzymes synthesized by hydrolysis will remain inactive [15]. Thrombin will stimulate fibrin to create a network to stabilize the platelets, which network will form a stable scaffold (thrombus) for the arrival of other substances attracted by the same platelets [16,17]. These substances are chemokines (bradykinins) and growth factors [14,17]. The known growth factors involved by platelets are platelet-derived growth factor (PDGF) with chemotactic and proliferative properties, basic fibroblast growth factor (bFGF) involved in angiogenesis processes, transforming growth factor beta (TGF-β) with the ability to influence the proliferative phase, and epidermal growth factor (EGF) to interact in the proliferative phase [16]. Resident immune cells (mast cells) will emit small amounts of hydrogen peroxide, attracting additional immune cells and histamine, and stimulating the next stage of inflammation [14,18]. Generally, the duration of the homeostasis phase is a few hours, depending on the area of the lesion involved and on the clinical subjectivity of the patient [10].

Repair phase: inflammation

The inflammatory phase overlaps with the waning phase of homeostasis, covering a time span of about a maximum of two weeks. The first inflammatory intervention begins a few minutes after the presence of the lesion, counteracting the possible invasion of pathogens [10]. Resident mast cells stimulate vascular permeability, thanks to the recruitment of histamine, bradykinin, leukotrienes, tumor necrosis factor-alpha (TNF-α), and interleukins (IL)-1,6 and IL8 [13,14]. Mast cells are stimulated to intervene by the activation of several receptors that the cells themselves possess, such as toll-like receptors, Mas-related G-protein-coupled receptor X2 (MRGPRX2), and high-affinity IgE receptor (FcεRI) [19,20]. The latter receptors are activated by pathogen-associated molecular patterns (PAMPs) [17]. This cascade mechanism will promote the action of nuclear factor kappa B by attracting other immune cells to the damaged area.

In this phase, damaged cells produce damage-associated molecular patterns, proteins that "feel" the damage of the metabolic environment in which they were released, alerting the immune system [21]. The first cells that respond are leukocytes; in particular, neutrophils. In the first 48 hours neutrophils act as prime immune actors [16]. Neutrophils kill the antigens present through proteases, with the oxidation they generate (free radicals or reactive oxygen species (ROS)), through various microbial substances and by creating extracellular traps with which to incorporate foreign substances [16]. Neutrophils orchestrate the early stages of phagocytosis and the secretion of further cytokines and chemokines [13,17]. If leukocyte action is effective, neutrophils undergo apoptosis or migrate to other sites (neutrophil reverse migration) [13,16]. Monocytes are the second immune cells called to the injured area and represent the beginning of the second inflammatory phase. Monocytes rapidly transform into macrophages with a high phagocytic capacity and pro-inflammatory activity [16]. They synthesize multiple substances that enhance the inflammatory process and defense components, such as IL-6, IL-8, IL-1beta, TNF-α, and monocyte chemoattractant protein-1 (MCP-1) [16,17].

Macrophages also synthesize matrix metalloproteinases (MMPs) to disassemble the extracellular matrix and prepare the tissue for other subsequent stages of healing [16]. If the inflammatory stimulation falls within a physiological context, pro-inflammatory or M1-type macrophages tend over time to transform into another M2-type or anti-inflammatory phenotype. M2 synthesizes cytokines such as transforming growth factor-beta 1 (TGF-β1), IL-10, and angiogenic substances such as vascular endothelial growth factor (VEGF), and other growth factors that will introduce the proliferation phase, such as PDGF and FGF2 [10,16,17]. Other substances are produced such as nerve growth factor (NGF), connective tissue growth factor (CTGF), and cysteine-rich 61 (Cyr61) [22]. The intervention of M2 favors the recall of keratinocytes, fibroblasts or fibrocytes, and cells that will become endothelium [16].

Repair phase: proliferation

The transition in this phase is characterized by fibroblasts transforming into myofibroblasts; the time frame of the proliferation covers about 30 days, with a first intervention 3-10 days after the advent of the lesion [13,23]. Fibroblasts residing in the dermis produce substances capable of synthesizing type I and III collagen, fibronectin, proteoglycans, and hyaluronic acid, which substances will fuse new extracellular matrix (ECM) [13]. The same fibroblasts can secrete Neu differentiation factor (NDF or neuregulin), which facilitates the recall of further keratinocytes [22]. The accumulation of keratinocytes is the first step of this phase so that re-epithelialization develops; the edges of the wound are filled and gradually a new covering is created, restoring the epidermis-dermis bond [16]. All the angiogenic factors secreted in the following phase (inflammation) find maximum freedom of action. Pericytes are recruited to form the same vascular volume before the injury and stimulate the angiogenesis phase [16]. The ECM and the new vessels constitute the granulation tissue or stroma, thanks also to the intervention of mesenchymal cells of different origins (follicles, nerve endings, bone marrow, fat, sebaceous glands) [13,22]. Another step of proliferation is fibroproliferation deriving from the action of fibroblasts. TGF-β1 and myocardin-related transcription factor-A intervention promote the transformation of fibroblasts into myofibroblasts (Myo) in granulation tissue, one to two weeks after injury [13,22,24]. Myo possesses α-smooth muscle actin, thanks to which they can generate a cyclic contraction and produce mechanical tension [25]. The contraction helps the new vascular tissue to form and further facilitates the work of the keratinocytes; the contraction closes the wound edges, at a rate of about 0.75 mm per day [13,22].

Repair phase: remodeling

In this stage, the granulation tissue transforms into scar tissue, with a time frame that can take over two years [17]. Many cells that have built the stroma undergo apoptosis or migrate to other tissues [22]. The ECM tends to reorganize itself, recompact itself, and reduce type III collagen, with an increase in type I, making the tissue more rigid, thanks to the coordinated intervention of MMPs, M2, and keratinocytes [16,17]. The orientation of the collagen is influenced by the mechanical tension that the tissue feels and undergoes [23]. The newly formed tissue is always weaker and less able to handle the mechanical information it receives, compared to the same tissue prior to the onset of the trauma [23]. The type I collagen fibers tend to be parallel, with a tensile capacity that reaches 20% (in the third week after the injury), up to a maximum strength expressed of about 80% (after many weeks), compared to healthy tissue [16,22]. If the metabolic mechanisms are functional, the scar becomes thinner, less red, and with fewer blood vessels [22].

Scarring

When a lesion involves deep trauma or involves a large area of skin, the healing processes leave a scar [22]. A mature scar is composed of about 90% type I collagen, while the remainder is type III; approximately 50% of total scar tissue is collagen [22]. The scar contains no sebaceous glands or follicles; the ECM is less elastic than healthy skin, due to the reduced number of elastin [22]. A non-pathological scar has typical characteristics, such as hyperpigmentation and greater lifting than the perimeter of the epidermis, but these traits tend to disappear over time. Before a scar matures, it can be a source of itching and pain, as well as a temporary limitation in joint movement [22]. Probably, when the lesion is large, a specific fibroblast with the Engrailed 1 gene is recruited; this fibroblast could be responsible for the deposition of collagen that creates scarring [26]. Fibroblasts in the presence of a large wound are influenced by the mechanical tension they feel and undergo. This sensitivity is mediated, in particular, by the activation of focal adhesion kinase (FAK), monocyte chemoattractant protein-1 (chemokine), and extracellular-related kinase (signaling molecule) [27].

Pathological scarring

Pathological scars are defined as such when they negatively affect a person's health and quality of life (psychic, social, working) [28,29]. We can recognize different pathological scars, such as hypertrophic scars, keloids, atrophic scars, and striae distensae (SD) or stretch marks.

Hypertrophic scars

The first news of an excess of cicatrization was recorded on an Egyptian papyrus of 1700 BC [30]. Hypertrophic scars are morphologically characterized as a cutaneous elevation, with respect to the healthy epidermis line; they can arise after about two months from the advent of the lesion, with a rapid growth for a further six months [30]. Basically, these scars regress over a few years, flattening out and without symptoms [30].

There are anatomical areas where hypertrophic scars can be found in a greater percentage such as the shoulders, the cervical area, the presternum area, and the joint area of the knees and hips [30]. There does not seem to be an incidence of findings based on gender but, rather, based on the patient's age, with a higher percentage in the second and third decades. It tends not to recreate itself after surgical excision [30]. Hypertrophic scars are red and may cause severe itching and pain on palpation or pressure (allodynia and hyperalgesia) [23]. We find an increase in cytokines specialized in inflammation, such as chemokines (CCL2, CCL4, CCL5, CCL7, CCL13, CX3CL1, CXCR4), and interleukins (IL-6, IL-1β, IL-4, IL-8, IL-13, IL-17, IL-22) [10]. Hypertrophic scars shows low levels of MMP-2 and MMP-9, favoring a non-physiological deposition of collagen and proteins [31,32]. If the wound follows non-physiological healing pathways, M2 could persist at a time of action; this could attract a greater number of fibroblasts, favoring an increase in the amount of myofibroblasts [14].

The number of mast cells in hypertrophic scars is higher than in non-pathological scars, activating signaling pathways that stimulate fibroproliferation, such as TGF-β/mothers against decapentaplegic (SMAD) [14,30]. During the pathological inflammatory phase, the levels of PDGF and insulin-like growth factors-1 (IGF-1) increase; the latter hormone could accumulate and inhibit apoptosis processes in the remodeling phase [30,33]. There appears to be an increase in hypoxia-inducible factor (HIF), which would stimulate hypoxia, fibrosis, and further inflammation [11]. In the proliferation/remodeling phase, there is an imbalance between storage and cleanliness. Accumulate proteins (fibronectins, periostins, fibrillins, laminins) and collagen in the ECM, increase angiogenesis, creating a dense and compact stroma [10]. The pro-fibrotic stimulation by different substances such as VEGF, PDGF, TGF-β, and CTFG continues, laying the foundations for an overstimulation of fibroblast activity [10]. The synthesized collagen is mainly of type III, a structure with little elasticity and with an entropic organization; the result is a mechanically hard fabric, but not very capable of withstanding tensile stresses for prolonged times [23]. Focal adhesion kinase (FAK) is a macromolecular complex, among which we find the integrin, important for the mechanotransductive signaling pathways between the ECM and cytoskeletal actin [16]. When a mechanical signal arrives (stretch, compression, etc.), FAK is phosphorylated and activates extracellular-related kinase (ERK) and monocyte chemoattractant protein-1 (MCP-1) recall; if the mechanical signal is recorded from altered tissue structures, the response will be an increase in fibrosis [34].

Another signaling pathway that is activated under mechanical load is the Wnt/β-catenin pathway complex (canonical pathway); probably, with an altered mechano-metabolic environment, when translocated to the cell nucleus (β-catenin) it stimulates further fibrosis [35]. The mechanotransducive system, involving the phosphatidylinositol 3-kinase (PI3K)/AKT/mammalian target of the rapamycin (mTOR) signaling pathway, seems to undergo an altered response in the presence of mechanical stimuli [16]. In particular, in keratinocytes and fibroblasts present in hypertrophic scars, this signaling pathway would stimulate these cells to produce a pro-fibrotic, inflammatory, and angiogenic environment [16]. Rho family guanosine triphosphate (GTP)ases (Rho, Rac, and Cdc42) are important pathways mediating metabolism and cellular function. A disturbed function of this communication pathway is found in non-physiological cicatricial processes; an inadequately managed mechanical stimulus stimulates an increased contraction of the Myos (contractures) [16,36]. Fibroblast ion channel disturbances, particularly for calcium (transient receptor potential TRP), facilitate the transition to the Myo, and increase the production of fibronectin and collagen. Dysfunction of Piezo1 protein channels in hypertrophic scar Myo could enhance wound contracture [16]. The hypersensitivity of these ion channels to mechanical stimuli favors a pathological environment of the scar.

Keratinocytes can contribute to the formation of hypertrophic scars. Notch signaling pathway acts on the cell via transmembrane ligands; in the hypertrophic scar, there is an increased percentage of this communication system, generating an increased formation of pro-fibrotic substances [10]. Keratinocytes can synthesize high mobility group box protein B1 (HMGB1), which stimulates the production of fibroblasts in hypertrophic scars, and favors an inflammatory environment [10]. An excessive mechanical stimulus, compared to the perceptive capacity of the tissue, will create a chronic pathological scar environment [13,22]. Hypertrophic scars have vertically organized blood vessels, with highly disorganized collagen bundles in the dermal layer; the amount of Myo is clearly higher than a non-pathological scar [30,37]. The nodules present in hypertrophic scars represent the grouping of Myo, which nodules can impede the correct movement of the surrounding tissues and be a source of pain [30]. The origin of the Myo of pathological scars does not derive only from the damaged dermis, but also from endothelial cells, adipose cells, and epithelial cells; they can derive from macrophages, from bone marrow cells [38]. Generally, the occlusion of the microvessels at the wound site highlights a phase of healing regression; hypertrophic scars present vessels in a high number, more dilated, and with a faster flow than non-pathological scars [18]. Hypertrophic scars has a more active and reactive vascular activity to mechanical stimulation; with increased permeability, the vessels facilitate the persistence of inflammation [18]. We must remember that local inflammation is always a systemic response [1].

Neurogenic inflammation

Neurogenic inflammation is defined as the local and systemic neurological response to neuropeptides from an inflammatory environment [39]. The skin has numerous afferents distributed in all layers, such as unmyelinated C-type fibers, autonomic-type fibers, and myelinated Aδ-type fibers [39,40]. Afferents sensitive to mechanical, nociceptive, and psychological stress stimuli synthesize peptides with neuromodulatory, neurohormonal, and neurotransmitter functions. The production of these neuropeptides affects the immune response of specialized cells (lymphatic cells and mast cells), and the activation of keratinocytes for the paracrine synthesis of pro-inflammatory cytokine substances [39,40].

Cutaneous afferents relate to the central and peripheral nervous systems, and the immune and endocrine systems [39]. When there is a stimulation that activates the skin receptors of the scars, the responses can be different. One of these responses is the production of substance P or neurokinin type 1 (NK-1), which binds to endothelial receptors (NK-1R), promoting vessel proliferation and permeability; NK-1 interacts with calcitonin gene-related peptide (CGRP), increasing the inflammatory status [39,41]. These mechanisms stimulate the activation of P-selectin (transmembrane protein) on endothelial cells and E-selectin (endothelial-leukocyte adhesion molecule 1), with the result of amplifying the inflammatory response and nociception [39]. CGRP has a great influence on vasodilation, amplifying its action and attracting inflammatory cells; collaborates with the transcription factor nuclear factor kappa-light-chain-enhancer of activated B cells (NF-kB). Stimulation of NF-kB by CGRP results in a stronger recall for pro-inflammatory cytokines and is probably one of the causes of the presence of pruritus and pain [40,42]. The psychological stress (and the neuroinflammation present) that patients feel if they undergo unwanted aesthetics or if the scar symptoms are a source of pain and itching can lead to an exacerbation of the skin inflammatory response. Adrenocorticotropic hormone (ACTH) released by psycho-physical stress stimulates the production of systemic cortisol, creating a closed circle. The stress suffered by patients can stimulate the synthesis of biochemical substances by cells located in the skin such as macrophages, keratinocytes, melanocytes, and fibroblasts/Myo; these substances will stimulate the production of cortisol, local inflammation, and pain, with a worsening of the emotional state [39]. These substances are glucocorticoids such as corticotropin-releasing factor, proopiomelanocortin, β-endorphin, ACTH, and melanocyte-stimulating hormones [39]. The presence of inflammation in pathological scars is neurogenic and has a local and systemic impact [1].

Keloids

**Figure 1:**
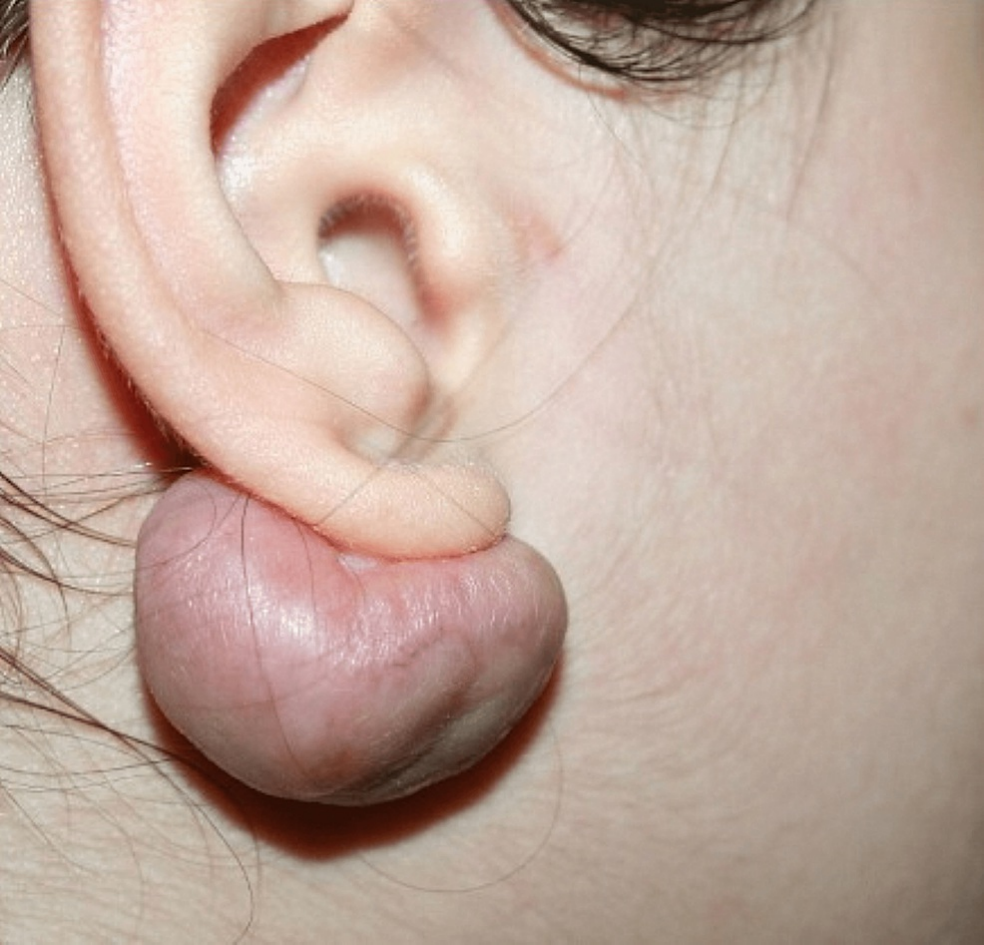
A keloid in an anatomical area typical for these lesions, following earring placement Image credit: Bordoni Bruno

Keloids can arise in a skin lesion after years, regardless of the size of the lesion (from large to small lesions, and from folliculitis), or develop from a hypertrophic scar. They rarely regress over time. If this event occurs, it affects the central area of the scar and after menopause in women [30,43]. On palpation, they have a moderate tightness, shiny surface, with a purplish or red color, or hyperpigmentation; there are no nodules, and the Myos are negligible [30]. There may be areas of ulceration, thinned epithelium, and often present with pain and itching; the boundaries of the keloids are irregular and can widen their surface, to the detriment of healthy skin, with a progression of decades [30,43]. The pain is often perceived in the central area of the keloids, while the itching sensation concerns the peripheral areas; phenomena of allodin and alloknesis can stimulate further pain and itching, respectively [43,44]. There are preferential anatomical sites for their appearance, such as the earlobe, chest, shoulders and scapular area, armpits, cheeks, upper limbs, groin area up to the genitals, the nipple area, and more rarely in other areas on the back and lower limbs and the penile area (Figure 1) [30,43].

Keloids recur if they undergo surgical debridement or simply undergo a biopsy [22,43]. Histologically they present type I and III collagen, and abundant elastin, compared to healthy skin with greater disorganization; they are more common in people with darker pigmentation and Asians, and individuals with a family history [22,43]. There is probably a pathological relationship between melanocytes and injured fibroblasts [22,43]. There is disagreement about gender preference, but there are more findings in the second and third decades of life and in adolescents, as well as during pregnancy [30,43]. In some cases, a genetic condition appears to exist (autosomal dominant but incomplete penetrance) [43]. There are congenital pathologies in which the appearance of keloids is favored, such as Rubinstein-Taybi, Goeminne syndrome, Bethlem myopathy, and a mutation affecting the absence of the cytoskeletal protein filamin type A [43]. Again, it seems that the higher presence of type E immunoglobulin is a favorable factor, as well as a higher number of mast cells, compared to healthy subjects; there would seem to be a relationship between keloids and type A blood [30].

The mechanical tension (skin stretching) that keloids undergo is a very important factor for their stimulation and proliferation, from the movement of the limbs and trunk to simple breathing [43,45]. Tension can generate an additional dermal inflammatory response at the site of injury; the direction of tension (a pull) is followed by healing. Similarly, constant tension felt and not properly managed, rubbing or the act of scratching the wound, worsens the response of the keloid [45-47]. Hypertension could be a risk factor for the development of keloids, as well as smoking, and the presence of rheumatism and osteoporosis, but the reasons are not known (perhaps related to systemic inflammation) [45,48]. Areas rich in sebaceous glands also seem to favor the onset of keloids [43].

Occasionally and for wounds present for several years, keloids may undergo suppuration, when the space of the hair follicle is occupied by cysts and the latter is trapped by scarring [43]. They are considered pseudo-tumors due to their growth and non-physiological reparative processes; it is a chronic and progressive disorder in the presence of neurogenic inflammation [43]. It is the inflammation process that could be the core of the keloids problem. It seems that there is a specific immune system response; perhaps the mechanical tension (skin stretching) that the keloids undergo is a very important factor for their stimulation and proliferation, from limb and trunk movement to simple breathing [43,45]. Tension can generate an additional dermal inflammatory response at the site of injury; the direction of tension (a pull towards the periphery) is followed by scarring. Similarly, constant tension felt and not properly managed, rubbing or the act of scratching the wound, worsens the response of the keloid [45-47]. An altered mechanical tension stimulates T-helper type 2 (Th2) cells; the latter stimulate greater production of interleukins (IL-4, IL-5, IL-6, IL-10, IL-13, IL-31) and TNF-α [30,49].

Keloids fibroblasts possess a higher number of receptors to respond to growth factors such as PDGF, TGF-β, and IGF-1, with persistence of their activity [30]. Keloids have an increased amount of MMP-2 with excessive ECM remodeling [30]. There is a large branching of microvessels and a higher quantity of hyalinized collagen, compared to hypertrophic scars and non-pathological scars [45]. Pruritus is an important problem for patients who have keloids. Mast cells in the dermis in keloids produce large amounts of histamine, nerve growth factor (NGF), and serine proteases, which facilitate fibroblasts to produce more collagen [49]. Periostin is one of the substances produced by fibroblasts; it is an itchy molecule that acts by activating the receptor of the integrin protein of the nerve fibers of the skin and this results in an increased synthesis of Th2 and pro-inflammatory cytokines [49]. Itching is a symptom of ongoing inflammation. The greater the sensation of itching, the greater the dysfunction of the cutaneous nerve endings, mimicking neuropathy [49]. Skin afferents, especially unmyelinated type C fibers, could express such neuropathy due to compression experienced by the excess of deposited collagen, mimicking a peripheral entrapment syndrome [49]. Cutaneous afferent fibers in keloids are longer and thinner (chronic distress hypoplasia) [49]. The neural suffering of the cutaneous afferent fibers generates a local response of synthesis of the neuropeptide substance P; the latter facilitates the survival of M2, fibroblasts, and mast cell degranulation, creating a closed loop of chronic neurogenic inflammation [49]. Likewise, NGF in keloids stimulates histamine production by keratinocytes, creating a closed loop that leads to itching and inflammation [49]. The epidermal layer is thicker with disorganization of the stratum corneum, making the lesion more sensitive to mechanical stress [50].

Clinical differentiation

There are indications in the literature to help the clinician differentiate between pathological scars. We can distinguish four endotypes of scars: (i) Stretched or flat, (ii) Contracted, (iii) Atrophic or depressed, and (iv) Raised [51]. The acronym that follows is S.C.A.R. As far as scar characteristics (phenotype) are concerned, they could be present in multiple endotypic identities.

Stretched scars are generally considered asymptomatic, linear, and with a color that reflects the patient's skin pigmentation, and are considered physiological [51]. The contracted lesions, particularly in burn patients, are often symptomatic (pain and functional limitation), and their symptoms depend on the area involved in the lesion (joint area, area of wide movements); they are conditioned by the mechanical tension they undergo [51]. Scars that sink below the skin line or are depressed or atrophic may be the imprint of a previous acne vulgaris [51]. Raised scars include hypertrophic scars and keloids; the latter are considered severe if the area they occupy is about 40 square centimeters or more [47].

The clinician will decide whether to carry out invasive (biopsies) and non-invasive evaluations (ultrasounds, cutometers and colorimeters, tomography, microscopy, spectroscopy), to differentiate any other pathologies; instrumental evaluations can be combined with numerical evaluation scales. Vancouver Scar Scale or Burns Scar Index is used to give a numerical value to the lesion (from zero to 13), before and after any treatment, by evaluating some parameters: vascularization, pigmentation, pliability, and scar height [52]. The Seattle Scale is based on the comparison of images taken of the patient and a 24-point numerical scale, evaluating some parameters such as the involved surface, thickness, pigmentation, and height [52]. Manchester Scar Scale (MSS) considers pigmentation, scar edges, texture, sheen, and distortion in shape, for a total of 18 points to be awarded; MMS is associated with a visual analog scale [52]. Hamilton Scale bases the assessment on vascularity, surface area, thickness, and pigmentation using photographs taken [52]. Patient and Observer Scar Assessment Scale observes pigmentation, vascularity, skin pliability, scar area, and patient symptomatology (pain and itch) [51]. Matching Assessment of Scars and Photographs is a photographic assessment (preferably with laser or three-dimensional photographs), taking into consideration pigmentation and color, thickness and height, surface area, and anatomical location [52]. Stony Brook Scar Evaluation Scale evaluates the thickness, the presence of elevation or depression of the scar, the color, the presence of sutures, and the general appearance; each assessment has a score ranging from zero to five [52]. The University of North Carolina's “4P” Scar Scale considers pain intensity, the presence of paresthesia, pruritus, and pliability, with a score ranging from zero to a maximum of 24 [52]. Patient-Reported Impact of Scars Measure scale evaluates the patient's perspective, with a lower score indicating less scar severity [51]. There is currently no scale or clinical tool (invasive and non-invasive) that considers all the endotypic and phenotypic characteristics of scars [53].

What manual medicine should consider

Currently, there is no gold standard of manual treatment to approach scars, but we can make some considerations, based on the information in the literature and reported in this article. Massage is probably the most used and prescribed manual form, even though there is no standardization and/or results, which are not always shared, with insufficient follow-up, both for hypertrophic scars and for keloids [10,31,54]. Equally, other manual treatments fall into this lack of evidence, such as lymphatic drainage, direct mobilization of the scar, myofascial relaxation, or induction, Kinesio taping, cupping, and dry needling; with these treatments, the lesion undergoes pressure and traction for a variable time [55].

The goals of these manual approaches are to improve pliability, vascularity, and elasticity, while reducing pain and itching. The studies do not always specify the type of scar (pathological or physiological), the number of patients is not high, and the working protocol is never (to our knowledge) repeated in subsequent studies [10,31,54,55]. The skin layers are always mechano-biologically active, and in case of injury, they undergo a sensory alteration [56]. This sensory alteration is not always well framed. The area of skin contralateral to the scar has sensory disturbances; moreover, pain could be caused by medullary areas subjected to constant nociceptive afferents with morphological alterations of the supramedullary centers [57].

Trying to resolve a painful sensation in a body system by working exclusively on the skin lesion area may not always lead to the desired benefits. It is not possible to know exactly and in real-time the behavior of the different components that make up the scar. By not respecting the needs of the skin (dermis and epidermis) when an undetermined intensity (pressure or stretching) is applied by the operator, inflammatory, pro-fibrotic phenomena, increased contracture, and further neurological disorders can be reproduced [10,13,16,22,23,31,34,36,43,45-47]. The elasticity of the skin does not depend on the manual approach, but on the processes that arise from the evolution of a lesion towards scar tissue; the skin will always retain an altered mechanical behavior, compared to the uninjured skin [14,22,23,30,37,43].

Manual techniques that seek increased vascularization of the pathological scar are in error. Scars considered pathological have a more developed and vasodilated vascular network, with greater reactivity to mechanical stimulation; stimulating the vessels means making an inflammatory status persist [18,45]. Injured skin is less able to withstand mechanical stimulation than healthy skin. As pointed out earlier, scars can become worse if subjected to minor trauma [14,22,31,43,50]. Applying any manual therapy that causes micro-injuries could be detrimental to the patient. It should be remembered that the tissue underlying a scar could be an adhesion, superficial or deep, involving soft tissue (muscles, viscera, vessels) or hard tissue (bones, joints). This means that the symptomatology of the lesion could derive from an underlying adherence and working only on the scar may not lead to an improvement in the symptoms.

**Figure 2:**
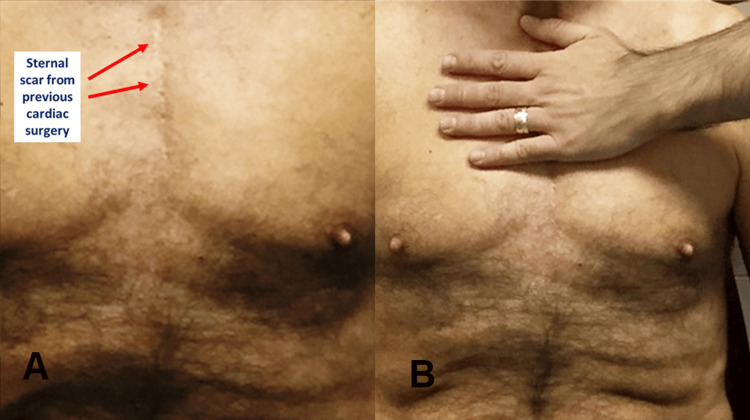
(A) Sternal scar from previous cardiac surgery in median sternotomy, with a time lapse since the operation of several years; (B) The operator's hand resting delicately on the scar, to apply an osteopathic approach with an indirect technique Image Credit: Bordoni Bruno

The question that arises is: Is it necessary to apply manual approaches? We know that there is a plethora of manual and instrumental, invasive, and non-invasive treatments available, but none have proven to be the standard preferred choice [58-60]. The clinician's goal is to facilitate the recovery of the psychophysical autonomy of the person who suffers from pathological scars, in concert with the patient's awareness [61]. The indication that emerges from the literature reveled that due to the hypersensitivity of injured tissues to mechanical stimulations, the most delicate approaches possible re to be used, avoiding treatments that are too direct and manually invasive. It is reasonable to consider any existing gentle manual approach as valid, only if the clinician in combination with a multidisciplinary staff deem a certain treatment procedure correct for the individual patient [62]. A very delicate approach that we can propose is an approach used in a previous randomized study with cardiac surgery patients: an indirect osteopathic technique, with the hand placed delicately and directly over the scar (Figure 2) [63]. The effectiveness would lie in the stimulation of the parasympathetic system. The cutaneous afferents reach the nucleus of the solitary tract of the vagus nerve and, through neural connections with different areas of the cortex and limbic areas, the immune system is stimulated to reduce its response. Furthermore, with a gentle approach, the parasympathetic system is able to elevate the pain threshold.

In a case report we described the same technique with the presence of an older scar, compared to trial patients with scars three to four days after surgery [64]. The operator does not induce any movement but follows the movement of the tissues that he palpates with his palm; the technique ends when the perceived movements appear in a homogeneous and non-chaotic pattern [63]. This therapeutic choice not only respects the "will" of the patient's tissue but is able to improve systemic inflammatory indices [65]. The approach is called "unwinding", conceived by Viola M. Frymann, DO; to deepen the topic, we recommend the work of Minasny [66].

Research should make further efforts to better identify which non-instrumental approach is most suitable for pathological scars. Experimental investigations should consider a larger follow-up, to truly understand the adaptation of the scar area, compared to the manual medicine approach. Furthermore, the clinical dermatologist should not base the therapeutic choices only on his own knowledge but also rely on other scientific figures.

## Conclusions

The skin layers are always mechano-biologically active, and in the event of injury, they undergo structural alterations that can negatively influence the function of the injured area and the neighboring anatomical areas. The lesion involving the dermal layer can resolve itself with the production of scar tissue, resulting in physiological or pathological scars. The latter are the source of symptoms that are not always easily identifiable or treatable, such as pain, itching, and important functional limitations. Currently, there is no gold standard of treatment for scars, either invasively or conservatively. One of the recommended conservative treatments is manual medicine, which acts directly on the scar. The article has reviewed the different biological phases of skin healing, both physiological and non-physiological, with the aim of highlighting the limits on which the manual approach is based and underlining the need to always individualize the treatment; the clinician and manual practitioner must remember that the scar is not just an area, but a system within the body system.
